# Intestinal microbiome gone native: gut microbiome shift and resistome diversity in first homecoming giant panda family

**DOI:** 10.3389/fmicb.2026.1737792

**Published:** 2026-03-26

**Authors:** Yuhang Wu, Linhua Deng, Xiangwang He, Dianyi Zhou, Shanshan Ling, Ming He, Qian Wang, Chengdong Wang, Minglei Wang, Honglin Wu, Linfeng Li, Desheng Li, Libing Yun

**Affiliations:** 1 Department of Forensic Pathology, West China School of Basic Medical Sciences & Forensic Medicine, Sichuan University, Chengdu, China; 2 China Conservation and Research Centre for the Giant Panda, Chengdu, China; 3 Key Laboratory of SFGA on The Giant Panda, Dujiangyan, Sichuan, China; 4 Sichuan Fire Research Institute of Ministry of Emergency Management, Chengdu, China

**Keywords:** 16S rRNA, antibiotic resistant genes, giant pandas, international travel, metagenomics, microbiome

## Abstract

**Introduction:**

The world-famous giant pandas (*Ailuropoda melanoleuca*) often travel abroad for public exhibitions and international scientific cooperations. Previous research has reported alternations in the gut microbiome structure and enrichment of gut antibiotic-resistant genes (ARGs) in human international travelers, the latter of which is harmful to native residents and the environment. The microbiome and ARGs of these animal travelers, however, have not yet been investigated, even though they often interact with local keepers, visitors, and other pandas.

**Methods:**

In this study, we have clarified the dynamic microbiome composition and snapshot of ARGs (resistome) of the first panda family returning from overseas. Fecal samples were gathered for high-throughput sequencing for both amplicon and metagenomics sequencing, which were collected on the first day of their quarantine (Admission stage) and 3 days after the quarantine (Release stage). Feces from two native captive pandas were used as controls.

**Results and discussion:**

The predominant *Escherichia–Shigella* proportion in the mother and father pandas decreased from 79.02 and 47.46% to 57.03 and 33.77%, while the *Streptococcus* abundance increased from 0.27 and 12.44% to 29.47 and 54.59%. The main genus of child pandas, *Weissella*, decreased from 45.24 to 0.02% after quarantine, and the *Streptococcus* ratio increased from 11.89 to 43.82%. Significant richness and bacterial diversities were found in these samples. The main ARG types are multidrug and polymyxin; the latter being an uncommon ARG in native pandas. Consequently, to protect local ecosystems from the introduction of novel ARGs, waste from translocated giant pandas should be managed under strict biosecurity protocols.

## 1 Introduction

The bamboo-fed giant panda (*Ailuropoda melanoleuca*), which harbors a carnivore gut microbiome (Xue et al., 2015), has attracted attention by global scientists. Genomic analysis suggested that no cellulase coding genes or any homologues were confirmed in the mammal’s genome (Li et al., 2010). The mystery of utilizing cellulose and hemicellulose in the staple food bamboo was hidden among the giant panda’s gut microbiome. A metagenomic analysis of their fecal samples brought out several members of the *Clostridium* genus containing putative digesting enzyme genes for cellulose and hemicellulose (Zhu et al., 2011). However, a majority of the bacteria within the *Clostridium* class were proven to be phylogenetically unrelated to known cellulolytic families, and none of them were found in other herbivores (Xue et al., 2015). Species from the *Escherichia*/*Shigella* genus and the *Streptococcus* genus dominate the gut microbiome in giant pandas (Xue et al., 2015). To elucidate the cellulose and hemicellulose-degrading capacity of these microbes, more in-depth metagenomic and metatranscriptomic approaches were carried out on the fecal samples from giant pandas, together with other herbivores, carnivores, and omnivores (Guo et al., 2018). The relative abundance of genes coding for cellulose and hemicellulose digestion enzymes in giant pandas was significantly lower than the corresponding genes in herbivores (horse, cow, sika deer, etc.) (Guo et al., 2018). For microbial gene functional profiling, Zhan et al. identified 7,134 genes by aligning the 724,019 non-redundant genes to the CAZy database, and these targeted genes encode 184 members of the glycoside hydrolase (GH) family (Zhan et al., 2022). The GH5 subtype, which was characterized as endoglucanase (EC 3.2.1.4) by Kyoto Encyclopedia of Genes and Genomes (KEGG), was found in the metatranscriptomic sequences of giant pandas (Zhan et al., 2022). The relative abundance of these pandas was approximately 0.94%. Zhan et al. also found that the endoglucanase-encoding genes identified in their research were primarily harbored in the genomes of *Paenibacillus elgii*, *Lachnoclostridium phytofermentans*, *Clostridium longisporum*, and *Cellulosilyticum lentocellum* (Zhan et al., 2022). The gut microbiome cannot provide strong support for the complete digestion of bamboos in giant pandas. Thus, the unique and special gut microbiome of giant pandas has become a focus area of research among researchers worldwide.

With the hardworking and persistent efforts of humanity, the conservation status of giant pandas was altered from “endangered” to “vulnerable” in 2016 (Swaisgood et al., 2016). Thus, they often interact with zookeepers, scientists, and tourists. Previous studies have interpreted that microbial diversity alteration (Worby et al., 2023) and that antibiotic-resistant genes (ARGs) increase (Cheung et al., 2023; Worby et al., 2023) in the gut microbiome of international human travelers. These travelers departed from their residences and lived at a different place for a period. For the gut microbiome of the 267 recruited American international travelers, Shannon diversity was decreased after travel in 61% of the volunteers (Worby et al., 2023). These American travelers attained multiple antibiotic-resistant genes after traveling with significant resistance to fluoroquinolone (Worby et al., 2023). The gut microbiome and resistome of 90 healthy Chinese adults in Hong Kong SAR, China, were not significantly altered after their international trip (Cheung et al., 2023). The number of antibiotic-resistant genes, such as *aadA*, *TEM*, and *tetR,* was notably enriched (Cheung et al., 2023). Another research reported that the gut microbiome of veterinary interns became similar to that of workers at a swine farm 3 months after the internship, following environmental exposure (Sun et al., 2020). One study also demonstrated that the accumulation of ARGs in international travelers poses potential risks to public health (D’Souza et al., 2021).

However, similar investigations of giant pandas returning from overseas have not been reported. Only the ARG profiles of native giant pandas were investigated and compared. It was reported that the abundance and richness of ARGs in captive giant pandas were significantly higher than those of wild pandas (Guo et al., 2019). The ARG composition of the gut microbiome in wild giant pandas from different locations also showed differences, as shown by the non-metric multidimensional analysis (Hu et al., 2021). In native pandas, an increase in antibiotic resistance—potentially driven by the frequent antibiotic treatments and possible anthropogenic ARG transmission—has been discussed (Deng et al., 2024). Therefore, it is necessary to characterize the resistome and microbial community of giant pandas returning from overseas to better inform conservation strategies and mitigate the risk of ARG dissemination to native captive populations and surrounding ecosystems. In this study, we reported the dynamic microbiome composition and resistome snapshot of the first returned panda family using their fecal samples using high-throughput sequencing.

## 2 Materials and methods

### 2.1 Pandas’ information

Five pandas, including the returning family (the panda pair and their offspring) and two native captive pandas, were involved in the study with the approval of the China Conservation and Research Center for the giant pandas. All giant pandas were sourced from the China Conservation and Research Center for giant pandas. The codename, age, and gender information are listed in Supplementary Table S1. Pandas 4 and 5 were chosen as controls to minimize the effect of distance because their territories are near but not adjacent to the area of the quarantined family. The three returning giant pandas were transported to the quiet environment shared with other captive-bred pandas immediately after disembarking. They underwent a one-month quarantine period during which they resided separately. All the food for these giant pandas, including the family and controls, is provided by the China Conservation and Research Center for the giant panda. All the diets were the same for the returned family and two controls. The health conditions of these pandas are excellent, and they have not received antibiotic treatment during the quarantine period.

### 2.2 Fecal sample collection

Fecal samples of all members of the panda family were collected from the first day of their quarantine to 3 days after the quarantine. Fecal samples from two native controls were collected on the third day after quarantine of the family. In total, 14 fecal samples were enrolled in our study. All samples were collected aseptically using sterile sample bags and then transferred to the lab wrapped in dry ice. Collection of samples compiled according to animal welfare requirements.

To compare the microbiome diversity of these pandas, two time points were selected, and samples collected at these periods were renamed. The first day of quarantine and 3 days after quarantine were set as Admission and Release stages. Three fecal samples were collected at the Admission stage and 9 fecal samples were collected at the Release stage. Two fecal samples from control pandas were set as controls. Samples from the control group were collected on the third day after quarantine in order to show the post-quarantine similarity of the gut microbiome of returning giant pandas. For the fecal samples collected at the Admission stage, we grouped them as S1, S2, and S3, which correspond to the Panda Codename in Supplementary Table S1. S1, S2, and S3 belong to the Admission group. Fecal samples collected at the Release stage were grouped as E1, E2, and E3, which constituted the Release group. Fecal samples from two controls were renamed as E4 and E5, but they were assigned to a different group called control (C).

Considering the small number of our obtained fecal samples, we designed biological repeats to improve the reliability of our research. For feces collected at the Admission stage, four biological repeats of each panda in the family were collected—three of them were used for amplicon sequencing and the remaining one was used for metagenomic sequencing. For feces collected at the Release stage from the family, only one biological repeat each day per panda was collected for amplicon sequencing. Four biological repeats of each control panda at the Release stage were collected—one repeat was for metagenomic sequencing, and the remaining three were used for amplicon sequencing.

The number of biological repeats at each stage is recorded in Supplementary Table S1.

### 2.3 DNA extraction

For 16S analysis, DNA of 24 fecal samples was extracted by ALFA-SEQ Advanced Soil DNA Kit (Magigene, Guangdong, China) following the manufacturer instructions of the kit. The concentration and purity of DNA were measured using the NanoDrop One (Thermo Fisher Scientific, MA, United States). For ARG analysis, the genomic DNA of four fecal samples was extracted by Guangdong Magigene Biotechnology Co., Ltd. (Guangzhou, China) using ALFA-SEQ Advanced Stool DNA Kit (Magigene, Guangdong, China) according to the manufacturer’s instructions. DNA integrity and purity were monitored on 1% agarose gels. DNA concentration and purity were measured using Qubit 3.0 (Thermo Fisher Scientific, Waltham, United States) and Nanodrop One (Thermo Fisher Scientific, Waltham, United States) at the same time.

### 2.4 16S rRNA gene full-length sequencing and Metagenomic sequencing

16S rRNA genes were amplified using specific primer (27F: AGRGTTYGATYMTGGCTCAG, 1492R: RGYTACCTTGTTACGACTT) with barcode. The PCR instrument was Bio-Rad S1000 (Bio-Rad Laboratory, CA, United States). The length and concentration of PCR products were detected by 1% agarose gel electrophoresis. PCR products were mixed in equidensity ratios according to GeneTools Analysis Software (Version4.03.05.0, SynGene). Then, the mixture of PCR products was purified using a HiPure Gel Pure DNA Mini Kit (Magen Biotechnology Co., Ltd., Guangdong, China). The sequencing library was obtained according to the 16S Amplification SMRTbell^®^ Library Preparation workflow. The library was sequenced on the PacBio Sequel II platform (Guangdong Magigene Biotechnology Co., Ltd., Guangzhou, China). The 16S rRNA gene sequencing yielded an average depth of 37015.92 reads per sample with a mean length of 1504.175 bp. NGS sequencing libraries were generated using the ALFA-SEQ DNA Library Prep Kit following the manufacturer’s recommendations, and index codes were added. The library quality was assessed on the Qubit 4.0 Fluorometer (Life Technologies, Grand Island, NY) and Qsep400 High-Throughput Nucleic Acid Protein Analysis System (Houze Biological Technology Co, Hangzhou, China). Finally, the library was sequenced on an Illumina NovaSeq 6,000 platform, and 150 bp paired-end reads were generated. The shotgun metagenomic sequencing produced an average of 12.46312 Gb of data (paired-wise), with an average of 83,087,448 reads (paired-wise) per sample.

### 2.5 Bioinformatics analysis and visualization

PacBio data processing, OTU cluster, and species annotation of 16S full-length rRNA genes were performed by Guangdong Magigene Biotechnology. Briefly, PacBio data were processed using the SMRT link (version 6.0) software, including data splitting, sequence error correction, and sequence format conversion. The clean data were clustered into operational taxonomic units (OTUs) based on a 97% similarity threshold with the UPARSE (Edgar, 2013) usearch software (V10).^1^ Chimera sequences and singleton OTUs were removed simultaneously using UPARSE. The annotation of taxonomic information for each representative sequence was obtained by mapping the 16S/silva_de_uncultured_All/v138^2^ database (Pruesse et al., 2007; Quast et al., 2013). The annotation confidence is 0.8. Finally, the OTU table and representative OTU sequences were processed by UPARSE, and the OTUs of chloroplasts, mitochondria, and unclassified archaea were removed.

The OTU table, taxonomy table, and OUT FASTA sequences were imported into R (version 4.4.2) (R Core Team, 2024) for diversity and statistical analyses. These analysis were performed using the phyloseq (version 1.50.0) (McMurdie and Holmes, 2013), ggplot2 (version 3.5.1) (Wickham, 2016), and vegan (version 2.6–8) (Oksanen et al., 2024) R packages. Prior to the analysis of microbiome composition and diversity, the OTU table from 24 samples was rarefied to the same depth by calculating the minimum OTU numbers of each sample. We found the top 10 most abundant genera of each sample and renamed the other genera as others for simplification. Principal coordinate analysis (PCoA) and unweighted pair group method with arithmetic mean (UPGMA) clustering analysis were applied using the Bray–Curtis distance matrix calculated from our samples. Linear discriminant analysis effect size (LEfSe) analysis was conducted on the Magigene Cloud Platform.^3^

The analysis of antibiotic-resistant genes (ARGs) was performed on four metagenomic sample data, ARG-OAP v3.0 (Yin et al., 2023). The pipeline was chosen because it was efficient and user-friendly. Before the ARG analysis, the raw metagenomic data were quality controlled by Guangdong Magigene Biotechnology using Trimmomatic (Bolger et al., 2014) v0.36 to acquire clean data. The parameters of Trimmomatic were set as follows: LEADING:3 TRAILING:3 SLIDINGWINDOW:5:20, MINLEN:50. The host genome sequences were removed from the raw data. The host genome can be downloaded from NCBI RefSeq assembly (GCF_002007445.2) or GenBank (GCA_002007445.3). To perform the ARG-OAP pipeline, we wrote a shell script and used the reads per kilobase per million mapped reads (RPKM) data of ARGs from main types to subtypes for further visualization.

### 2.6 Statistical analysis

The non-parametric test and the Kruskal–Wallis test were applied in our samples to assess the differences in Shannon diversity (*p* < 0.05) because of the small sample size in our study. The significance of the beta diversity matrix was evaluated using the permutational multivariate analysis of variance (PERMANOVA) test with a *p* < 0.05. Statistical tests and plot visualizations were performed using R scripts and Python scripts.

### 2.7 Data availability

The raw sequencing data including 16 s full amplicon and metagenomic sequencing data are accessible from the NCBI Sequence Read Archive with the accession number PRJNA1120402.

## 3 Results

**Figure 1:**
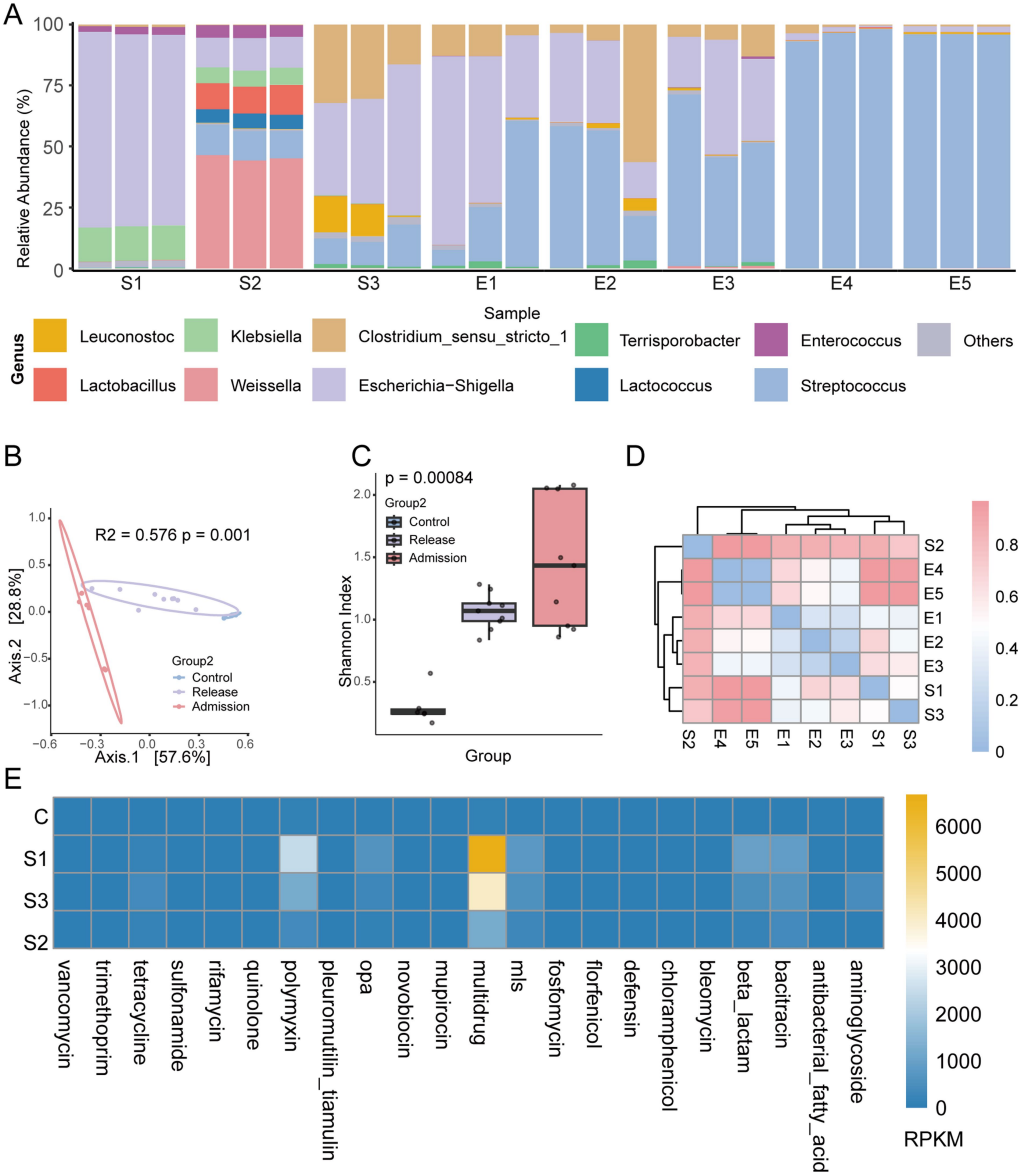
Microbial diversity and ARG diversity of the quarantined giant panda family. **(A)** Relative abundance plot of the top 10 genera for each sample. The pandas involved in this study were named numerically. Quarantined panda family: mother (1), child (2), and father (3). Native captive pandas: 4 and 5. S1–S3: fecal samples of pandas 1–3 collected at the admission stage (admission) of quarantine. E1–E3: fecal samples of pandas 1–3 collected at the release stage (release) of quarantine. E4 and E5: control group, fecal samples of pandas 4–5 collected at the release stage (release) of the quarantine. **(B)** PCoA plot of Bray–Curtis distance of all samples. PCoA: principal coordinates analysis. Admission: admission stage of the quarantine. Release: release stage of the quarantine. Control: control. **(C)** Box plot of Shannon diversity of all samples. PCoA: principal coordinates analysis. **(D)** The UPGMA clustered heatmap of Bray–Curtis distance of all samples. UPGMA: unweighted pair group method using arithmetic mean. **(E)** ARG abundance heatmap at the admission stage of quarantine of the family. *Mls*: macrolide–lincosamide–streptogramin. *Opa*: other_peptide_antibiotics. RPKM: reads per kilobase per million mapped reads.

Fecal samples of three members of the family were collected since the first day of their quarantine. Giant panda information and details of sample collection are described in Supplementary Table S1. The predominant genus in fecal samples from all adult pandas (S1, S3, E1, E3, and C) and panda child (E2) included *Escherichia–Shigella*, *Streptococcus,* and *Clostridium sensu stricto 1* (Figure 1A), which is consistent with previous reports (Xue et al., 2015). The relative abundance of *Escherichia–Shigella* and *Streptococcus* accounted for 29.26 and 18.76% of their total sequences (Xue et al., 2015). *Clostridium* is also in the top 10 genera reported in their results (Xue et al., 2015). However, the proportion of these genera varies among samples collected at different quarantine stages and derived from various hosts. The abundance of *Escherichia-Shigella* in the panda mother (No.1) and father (No.3) decreased from 79.02 and 47.46%, respectively, in the Admission stage (Admission, S1 and S3) (Table 1) of quarantine to 57.03 and 33.77% in the Release stage (Release, E1 and E3) (Table 1). Richness of *Escherichia-Shigella* in the panda child (No.2) increased from 12.74 to 28.41% during quarantine (S2 to E2) (Table 1), whereas the proportion of *Streptococcus* in all family members increased over the same period (Table 1). The percentages of *Streptococcus* at the Admission stage are 0.27% (mother), 11.89% (child), and 12.39% (father) (Table 1). At the Release stage, the corresponding percentages increased to 29.47% (mother), 43.82% (child), and 54.59% (father) (Table 1). Despite a predominant genus shift during the quarantine, the specific genus of each family member was also found on the first day of their quarantine (S1–S3). *Klebsiella* was predominant in the panda mother (14.11%) and child (6.67%), while *Clostridium* was rich in the panda father (26.37%) (Supplementary Table S2). *Weissella* was the most abundant genus in the panda child (45.24%) (Table 1), accompanied by two rich genera, *Lactobacillus* (11.34%) (Supplementary Table S2) and *Lactococcus* (5.83%) (Supplementary Table S2). Interestingly, these three genera belong to lactic acid bacteria, particularly *Weissella* and *Lactobacillus,* which are often isolated from fruits and vegetables (Fessard and Remize, 2019).

**Table 1:** Predominant bacterial genera percentage of all pandas at two stages.

Sample	Streptococcus	Escherichia-Shigella	Clostridium_sensu_stricto_1	Weissella
S1	0.0027	0.7902	0.0092	0.0003
S2	0.1189	0.1274	0.0029	0.4524
S3	0.1244	0.4746	0.2637	0.0008
E1	0.2947	0.5703	0.1014	0.0004
E2	0.4382	0.2841	0.2217	0.0002
E3	0.5459	0.3377	0.0816	0.0084
C	0.9575	0.0211	0.0125	0.0005

Quarantined panda family: mother (1), child (2), father (3). Native captive pandas: 4 and 5. S1–S3: fecal samples of pandas 1–3 collected at the Admission stage (Admission) of quarantine. E1–E3: fecal samples of pandas 1–3 collected at the Release stage (Release) of quarantine. C: control group, fecal samples of pandas 4–5 collected at the Release stage (Release) of the quarantine.

To compare alpha and beta diversity, these samples were divided into three subgroups. Fecal samples collected from the family on the first day of quarantine were assigned to the Admission group (S1–S3). Samples obtained at the Release stage of the quarantine were designated as the Release group (E1-E3). Samples from the two additional native captive giant pandas at the Release stage were regarded as the control group (C). The Shannon index of the control group was significantly different from that of the Admission group and Release group (Figure 1C) (P = 0.00084, 95% confidence intervals). Principal Coordinate Analysis (PCoA) analysis showed that samples within each group, respectively, formed clear clusters exhibiting similarity, and the three groups were distinct, separate from one another (Figure 1B) (R2 = 0.576, *p* = 0.001, 95% confidence intervals). Nevertheless, samples from S2 were kept away from S1 and S3 in the cluster tree (Figure 1D). This remote separation agrees with the different genus composition of S2 in Figure 1A. LEfSE analysis revealed that *Escherichia–Shigella*, *Clostridium sensu stricto 1,* and *Streptococcus* are three group-specific species in all samples (Supplementary Figure S1a).

Additional findings demonstrated the ARG diversity among the panda family and native captive pandas. In particular, it was noted that multidrug ARGs were the most abundant ARGs in all family members compared with the native ones (Figure 1E). Multidrug resistance ARGs have also been reported as the main type of antibiotic-resistant genes in native captive (Zhu et al., 2021) and wild pandas (Jin et al., 2023). The second most ARG type in the family was the polymyxin ARG type, which was enriched in the two adults, although this type was not commonly carried by native pandas (Figure 1E). Further analysis of the subtypes of polymyxin ARGs revealed that four subtypes (*arnA*, *eptA*, *pmrF,* and *ugd*) were enriched in the family (Supplementary Figure S1c). The enrichments of multidrug and polymyxin ARGs occurred primarily in the two adult pandas in the family; with the mother exhibiting a higher overall abundance of ARGs than the father (Figures 1E; Supplementary Figures S1b–c).

## 4 Discussion

In our study, an obvious microbial composition was recorded in the quarantined panda family. The gut microbiome of quarantined panda family resembled that of native after their quarantine. The composition of the gut bacterial community could be influenced by several factors such as diet, aging, and antibiotics (Park et al., 2021). Further in-depth analysis should be performed to find out the mechanism of this predominant bacterial genus shift. It is noteworthy that the dominant bacterial genera of the panda child at the Admission stage were different from those of other pandas. The three most abundant genera, including *Weissella*, *Lactococcus,* and *Lactobacillus,* are lactic acid bacteria capable of producing lactic acid. These bacteria showed a minimum abundance in native young pandas (age 3–7 years) (Liu et al., 2023). However, the authors found high abundance of *Lactobacillus* in the panda cubs (age 1.1–2 years) and speculated that the richness of *Lactobacillus* was associated with milk feeding (Liu et al., 2023). The child panda in our research was older than 3 years and thus was not mild-fed. Another source of these species may be the food consumed by the child, as *Weissella* and *Lactobacillus* are often isolated from fruits and vegetables (Fessard and Remize, 2019). Further investigation into their food intake during the quarantine is warranted. Besides diet and age, another factor that can affect the gut microbiome of humans and animals is stress. Gut microbiota dysbiosis among frontline healthcare workers during the COVID-19 pandemic persisted for more than half a year after they experienced psychological stress during the initial outbreak of the pandemic (Gao et al., 2022). The gut microbiome of posttraumatic stress disorder model mice was disrupted compared to that of control mice in terms of richness and diversity (Yadav et al., 2023). More interestingly, the beta diversity in the female Brandt’s Voles’ gut microbiome has been significantly affected by chronic exposure to predators’ fecal odor (Gu et al., 2025). These findings underscore the importance of preventing external stress for our returning giant pandas. The three returning giant pandas were transported immediately after disembarking to the quiet base shared with other captive-bred pandas.

The other finding of this study is the new ARGs, polymyxin ARGs, found in two adults in the family. These ARGs were analyzed using the fecal samples collected on the first day of their quarantine and could therefore reflect the common intestinal microbiome and resistome established during their overseas lives. It has been reported that animals may take in ARGs from the polluted environment and foods (Sun et al., 2020; Huang et al., 2022). To identify their possible origins, we reviewed studies on polymyxin ARGs in the country where the panda family had lived for a long time. However, these ARGs have not been reported as dominant in that region. Another ARG type detected at high abundance in these returning giant pandas was macrolide–lincosamide–streptogramin (mls). This ARG class is also commonly observed in captive giant pandas (Deng et al., 2024). Previous studies have reported that the high relative abundance of mls was found in livestock wastewater (Zhu et al., 2025) and animal manure-amended soils (Zhu et al., 2022). Increased international collaboration is necessary to determine the sources of these ARGs. These unique ARGs are potentially harmful to native people and pandas because they may increase the environmental ARG burden. Therefore, careful disposal of waste from traveling giant pandas are recommended to prevent the spread of new ARGs.

This study has several limitations that should be acknowledged. First, the sample size of the control group was small, and the age and gender distribution of the control group are not representative. According to recent reports, the number of pandas living abroad is 56 (Global Times, 2024), and the total population of captive ones will hit 757 in 2024 (Huang and Peng, 2024). The population of wild giant pandas is approximately 1,900, which has increased by 73% since 1980 with continuous human effort (Lan et al., 2024). Two conclusions came from these reported numbers. First, the total population of giant pandas is still low. Second, the number of overseas pandas only accounts for a small part of the total population. The number of pandas involved in our research is so limited compared with studies focusing on native giant pandas. Besides, the three giant pandas involved in our research are exceptional even among all overseas ones. The two adult pandas had resided abroad for more than 20 years, and they had given birth to a baby during their long overseas residency. Their child also lived abroad for 3 years. To our knowledge, this represents the first case of a whole giant panda family returning to their motherland. Long duration required to collect fecal samples from these returning giant pandas because their return is not a common phenomenon. An ideal control group should include pandas of different ages and genders. However, the living environments of the pandas in the base are unique in China. Each panda has its own independent territory within the facility. To minimize the environmental impact of the experiment, we selected two pandas from the control group whose territories are closest to those of the experimental group. Unlike laboratory-reared mice, giant pandas are national treasures of China. Each panda in the facility has a private residence and typically does not cohabit with other pandas. Since the dynamic gut microbiome changes in returned pandas were discovered, we aimed to determine how similar the gut microbiome of post-quarantine pandas was to that of native captive pandas. We, therefore, compared microbial diversity between control panda fecal samples collected on the third day after quarantine and consecutive fecal samples from returning pandas collected over 3 days post-quarantine. The two pandas selected as controls had lived locally for extended periods and consumed diets indistinguishable from those of other pandas. Second, the sources of the newly identified polymyxin ARGs in our study have not been clarified. We reviewed the prevalence of ARGs in the habitats abroad where the three returning giant pandas reside and did not find any published reports indicating that polymyxin ARGs are dominant. To thoroughly investigate the sources of these ARGs, increased international collaboration is necessary.

This study is a primary report about the dynamic microbiome shift and resistome comparison of the homecoming pandas. Significant differences in the microbial population and antibiotic-resistant genes between the returning pandas and native pandas were discovered using our collected fecal samples. To our current knowledge, the giant pandas in our study represent a unique population. This represents the first known global instance of a family that resided abroad long-term and, after reproducing, returned to China as a complete family. The parental pair lived abroad for more than 20 years, and their offspring was born overseas and resided there for 3 years. The feces of returning pandas are very valuable for researchers and veterinarians to assess and compare the health conditions of these pandas. More resources, intelligence, and international cooperation are needed to unearth their metagenome, resistome, and virome in the future.

## 5 Conclusion

Finally, our main conclusions are: The predominant bacterial genera of returning giant pandas shifted from *Escherichia-Shigella* to *Streptococcus* after 1 month of quarantine. Uncommon polymyxin-resistant genes were enriched in the fecal samples of the homecoming pandas on arriving day. These ARGs may be hazardous to native captive pandas, their staff, and visitors.

## Data Availability

The datasets presented in this study can be found in online repositories. The names of the repository/repositories and accession number(s) can be found at: https://www.ncbi.nlm.nih.gov/, PRJNA1120402.

## Footnotes

1
http://www.drive5.com/usearch/

2
https://www.arb-silva.de/

3
http://cloud.magigene.com/

## Supplementary material

SUPPLEMENTARY FIGURE S1 LEfSe analysis results and ARGs abundance heatmap. (A) LDA scores of fecal samples from three groups. Admission: Admission stage of the quarantine. Release: Release stage of the quarantine. C: control. (B) Abundance heatmap of multidrug and polymyxin resistant ARG types. (C) Abundance heatmap of all polymyxin-resistant subtypes. LEfSe: Linear discriminant analysis Effect Size.

SUPPLEMENTARY TABLE S1 Pandas information and fecal samples collected during the quarantine.

SUPPLEMENTARY TABLE S2 Top 10 bacterial genera percentage of all pandas at two stages.
