# Supplementary figures and images for: Intestinal microbiome gone native: gut microbiome shift and resistome diversity in first homecoming giant panda family
Source: Front Microbiol. 2026 Mar 26;17:1737792. doi: 10.3389/fmicb.2026.1737792 (PMC13062235; doi:10.3389/fmicb.2026.1737792)

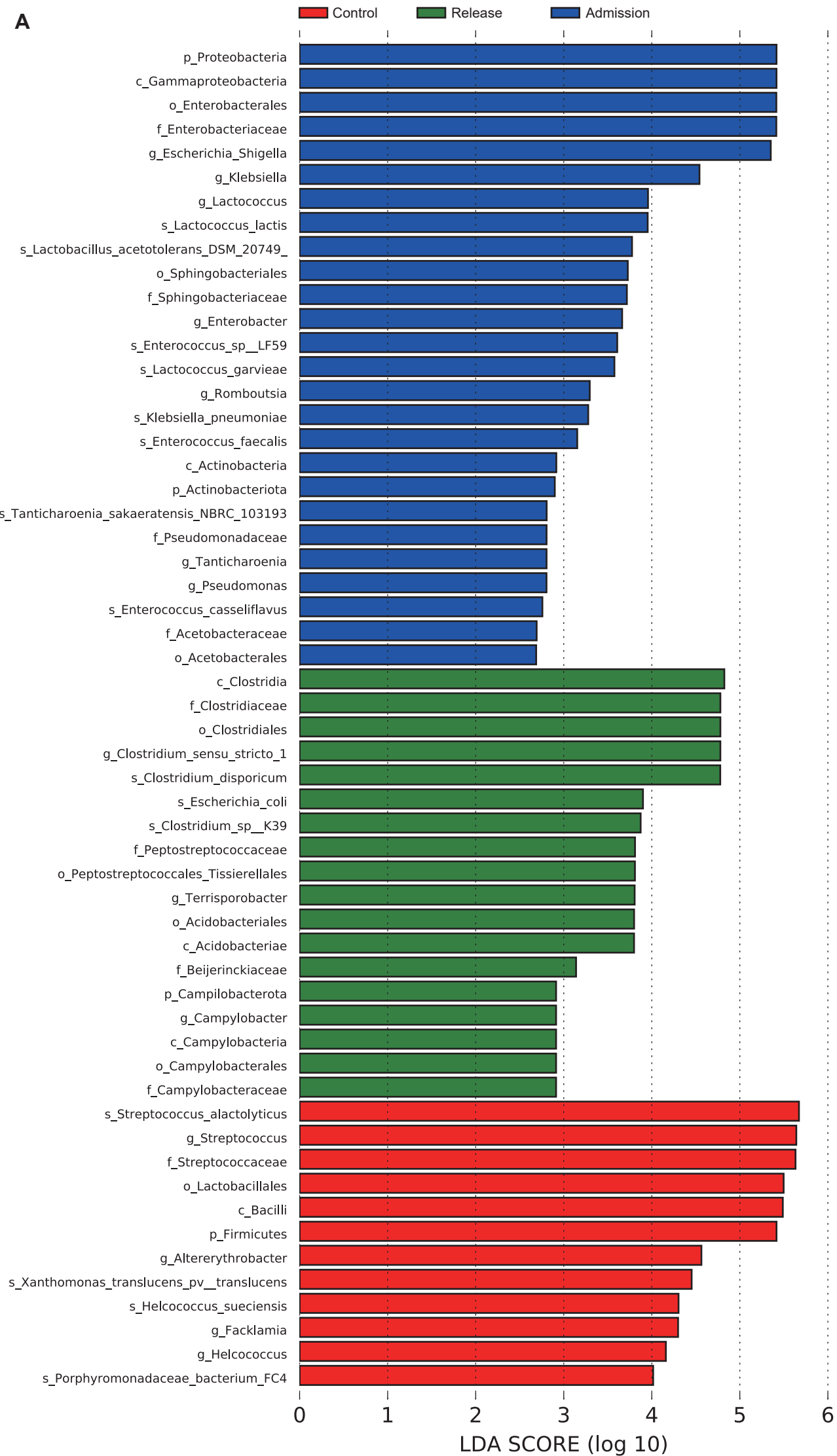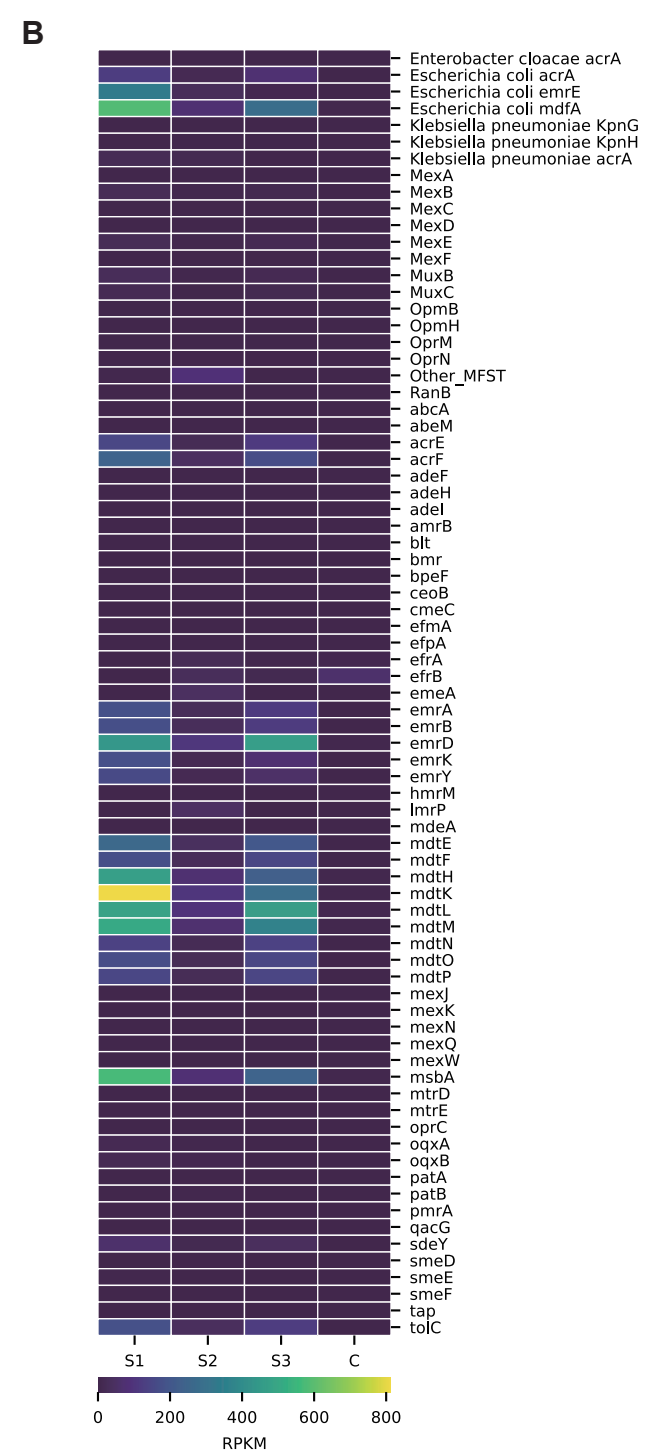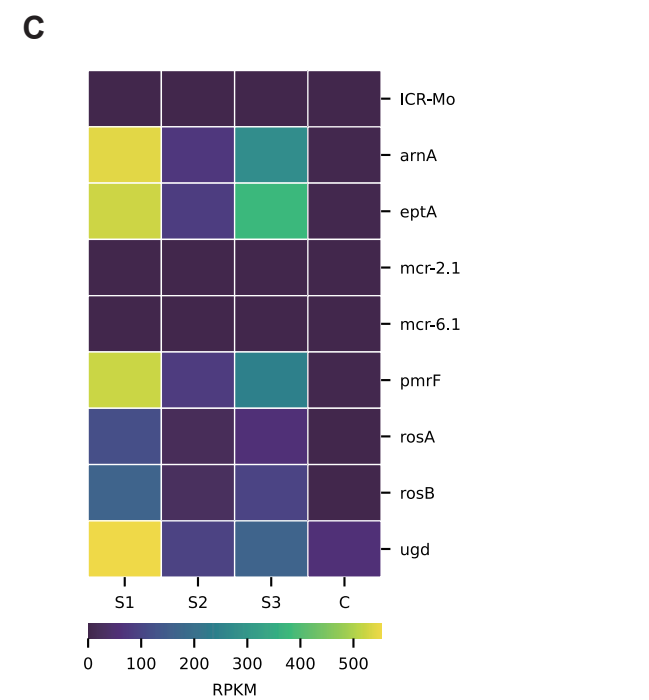

Supplement: SUPPLEMENTARY FIGURE S1 — LEfSe analysis results and ARGs abundance heatmap. (A) LDA scores of fecal samples from three groups. Admission: Admission stage of the quarantine. Release: Release stage of the quarantine. C: control. (B) Abundance heatmap of multidrug and polymyxin resistant ARG types. (C) Abundance heatmap of all polymyxin-resistant subtypes. LEfSe: Linear discriminant analysis Effect Size. [file Image_1.pdf]
